# A simplified workflow with end-point validation of real-time electrical cell-substrate impedance sensing of retinoic acid stimulated neurogenesis in human SH-SY5Y cells in vitro

**DOI:** 10.1186/s13104-023-06369-0

**Published:** 2023-06-01

**Authors:** Julia Joos-Vandewalle, Vanessa Steenkamp, Earl Prinsloo

**Affiliations:** 1 Biotechnology Innovation Centre, Rhodes University, P.O. Box 94, Makhanda, 6140 South Africa; 2 Department of Pharmacology, Faculty of Health Sciences, University of Pretoria, Private Bag X323, Arcadia, 0007 South Africa

**Keywords:** Electric cell-substrate impedance sensor, Neurogenesis, Differentiation, Neurite outgrowth, SH-SY5Y neuroblastoma

## Abstract

**Objective:**

Retinoic acid (RA) is known to transition proliferating SH-SY5Y neuroblastoma cells towards functional neurons. However, the activity of RA is restricted due to its photolability where any findings from prolonged time course observations using microscopy may alter outcomes. The aim of the study was to establish a real-time, long-term (9-day) protocol for the screening of differentiation events using Electrical cell-substrate impedance sensing (ECIS).

**Results and discussion:**

A differentiation baseline for SH-SY5Y cells was established. Cells were seeded and exposed to repeated spikes of RA using the xCELLigence real-time cell analyser single plate (RTCA-SP) for real-time monitoring and identification of differentiation activity over a 9 day period in order to be more representative of differentiation over a prolonged timeline. Specific features associated with differentiation (growth inhibition, neurite outgrowths) were confirmed by end-point analysis.

RA-induced growth inhibition and assumed phenotypic changes (i.e. neurite outgrowth) were identified by the xCELLigence analysis and further confirmed by end-point metabolic and phenotypic assays. Change in cellular morphology and neurite outgrowth length was identified by end-point fluorescence detection followed by computational analysis. Based on this it was possible to identify SH-SY5Y phenotypic differentiation with distinct phases observed over 9 days using Electric cell-substrate impedance sensing (ECIS) cell index traces providing a path to application in larger scale neurotrophic factor screening using this scalable technology.

**Supplementary Information:**

The online version contains supplementary material available at 10.1186/s13104-023-06369-0.

## Introduction

The SH-SY5Y human neuroblastoma cell line, a model of neuronal differentiation, provides a key tool for the screening and identification of novel compounds and the role they may play in driving neurogenesis [1]. Small molecule compounds are easily synthesized in high yield and purity and are thus ideal candidates for ultimate tissue regenerative therapies [2]. Warashina and co-workers demonstrated how a synthetic small molecule, neuropathiazol, selectively induced the neuronal differentiation of adult hippocampal cells [3]. Whether toxicity testing or therapeutic discovery, the balance between traditional end-point and real-time analysis needs to be established [4]. Due to cost, ease of application and analysis, early stages of drug discovery are highly reliant on end-point assays despite the overabundance of technologies that allow real-time monitoring of cellular differentiation e.g. live-cell microscopy and high content screening and analysis [5, 6]. End-point analysis may be robust and have the ability to scale, but much data may be lost during the experimental time course. In some cases the drawback of real-time monitoring is often the fact that appropriate fluorescence reporters may be needed and indeed inappropriate experimental design or the fluorophores themselves may alter or obscure outcomes [7–9]. This may be circumvented using label-free holotomographic microscopy and quantitative phase imaging [10].

Electric cell-substrate impedance sensor (ECIS) technology to monitor cellular activity allows for real-time monitoring of adherent cell proliferation, migration and morphogenesis in two and three dimensional assays [11–14]. Commercial systems based on the principle allow for cell seeding into single-use 96-well plates containing micropatterned gold sensing microelectrodes (e.g. the xCELLigence E-plate 96). Effectively, cell-attachment and contact with the microelectrodes increases resistance and hinders the current, resulting in continuously generated impedance values over time [14]. The impedance value obtained is label-free and directly corresponds to the number of adherent cells allowing for real-time reporting of cell viability over an experimental time-course [7]. The principle has been applied with varying success to 3D cell culture allowing for more realistic feedback of cellular responses. Commercial ECIS systems (xCELLigence) have previously been employed to monitor cellular differentiation [13, 15], specifically neural differentiation using SH-SY5Y [16], as well as human mesenchymal stem and cancer cell models [13, 17]. These studies typically proceeded over short time periods representing the initial phases of commitment in differentiation programming. The aim of this study was to establish a real-time, long-term (9-day) protocol for the screening and identification of novel small molecule compounds that are able to induce or enhance SH-SY5Y differentiation and subsequently aid in the development of synthetic neurotrophic factors.

## Methods

### Cell culture maintenance

SH-SY5Y CRL-2266^™^ cells (American Type Cell Culture, Manassas, USA) were grown in a basal medium of Dulbecco’s Modified Eagle Medium (DMEM):Hams F12 (1:1) containing 15 mM HEPES and L-Glutamine (Lonza, Basel, Switzerland), supplemented with 10% fetal calf serum (FCS ( v/v); BioWest, Nuaillé, France), 100 U/mL penicillin, 100 µg/mL streptomycin and 12.5 µg/mL amphotericin (PSA (v/v); Lonza). Flasks were incubated at 37 °C in a humidified incubator (Healforce, Shanghai, China) with a 5% (v/v) atmosphere.

Monitoring cellular proliferation and differentiation using xCELLigence real-time cell analyser.

**Fig. 1:**
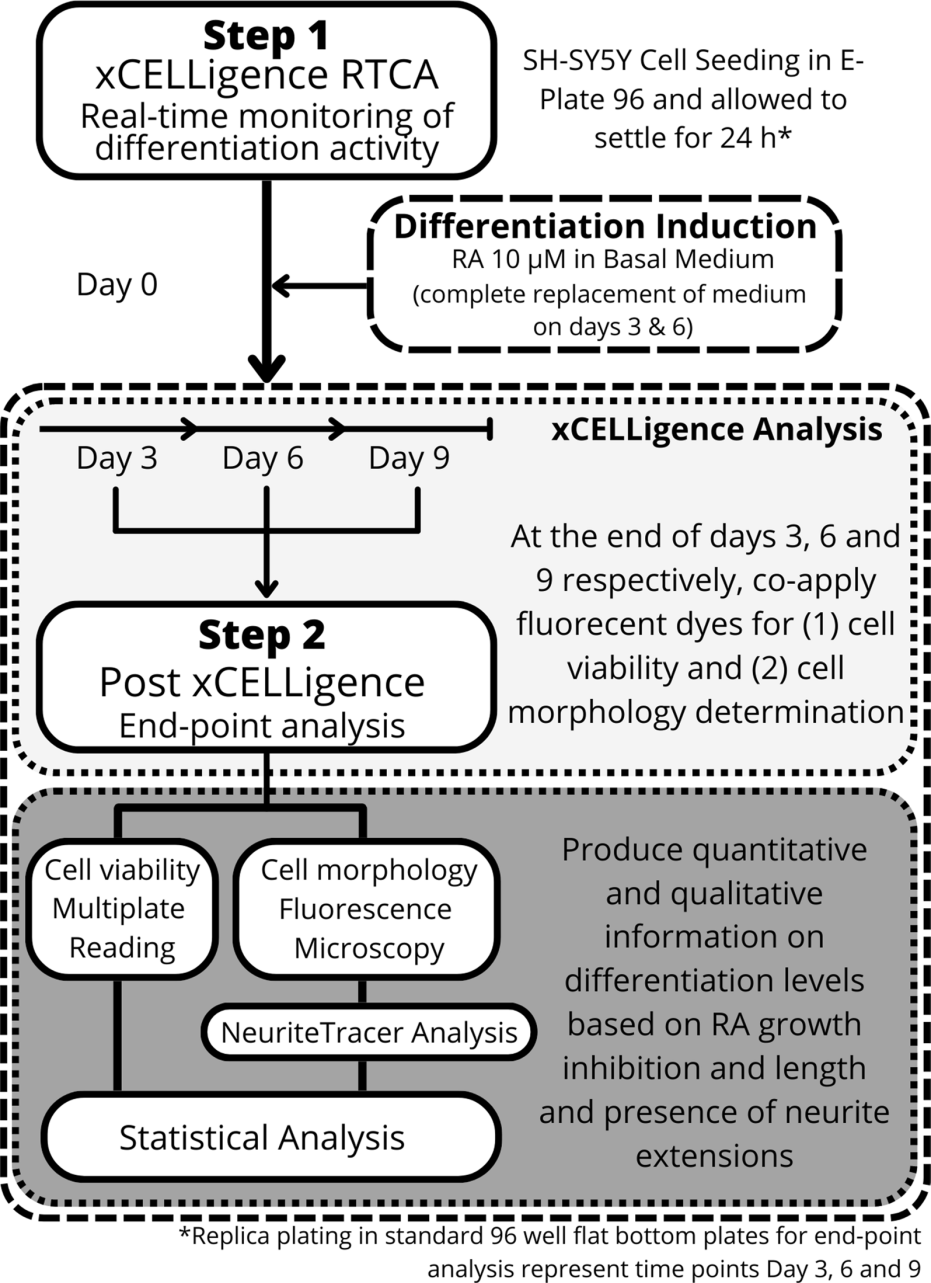
A two-step workflow, coupling real-time and end-point analysis system, for the monitoring of SH-SY5Y neuronal differentiation, as a lab-based tool for identification of drug candidates. Retinoic acid-induced differentiation was defined as the base-line control of differentiation.

The xCELLigence real-time cell analyser single plate (RTCA-SP) station was placed in a humidified CO_2_ incubator for 24 h to stabilize. Viable cell enumeration was performed following standard trypsinization using trypan blue staining and a haemocytometer to obtain an accurate count prior to the plating of cells. Following cell enumeration, 100 µL of pre-warmed basal medium was added to each respective well of the E-plate and a background measurement obtained before the plating of 10 000 cells/well in 50 µL medium. Cells were allowed to settle for 24 h post-seeding before initiating differentiation using 10 µM retinoic acid (RA, containing 0.01% dimethyl sulfoxide (DMSO) solvent (v/v); from a 1 mM stock concentration of 100% DMSO (v/v) stored at − 80 °C) to basal medium [18]. Due to the photolability of RA [19], all procedures were conducted under low light conditions and replenished every 3 days over a total period of 9 days by removal of media and replacement with fresh media at a total volume of 150 µL. To account for cellular metabolism[18, 20–22], RA concentrations were replenished every 3 days to ensure maintained differentiation programming (Fig. 1 Step 1).

Cell monitoring was undertaken with readings being captured with 15 min sweeps using the xCELLigence RTCA software (v 1.2.1 1002). The days were programmed as an initial 24 h step, followed by three 96 h steps in the controller software to pause the readings during media changes.

### End point fluorescence monitoring of differentiation

To confirm changes in cellular proliferation following addition of RA, the WST-1 assay (Roche) was employed as per manufacturer’s instructions. Cells were seeded at 10 000 cells/well in a 96-well plate and allowed to settle for 24 h before being treated with RA at 10 µM. An untreated control and DMSO (0.01%) vehicle control wells were included for comparison. Following 96 h (Day 3) incubation, spent media was removed and replaced with 110 µL pre-warmed fresh basal media, containing 1:10 dilution (v/v) of WST-1 reagent and incubate for 2.5 h at 37 °C in a humidified CO_2_ incubator. Absorbance at 450 nm (reference wavelength of 650 nm) was determined using a SynergyMx multiplate reader (BioTek). The average of three consecutive readings were recorded. Wells containing media and WST-1 reagent only were used as a blank and subtracted from all data obtained. This was repeated for plates incubated to Day 6 and Day 9. Each experiment was carried out on three occasions, in duplicate (n = 6).

End point analysis was performed using 96-well plates run in parallel to the xCELLigence system in the same incubator. Sufficient replica plates were prepared to allow end point analysis at 3, 6 and 9 days posttreatment (Fig. 1 Step 2). Flat bottom, sterile 96-well plates (Nunc^™^, USA) were prepared and treated as described in the section above. At the relevant time points, the 96-well plate was removed for fluorescence analysis. Fluorescence monitoring of neurogenesis was achieved using the Neurite Outgrowth Staining Kit (Life Technologies, Carlsbad, California, USA) as per manufacturer’s instructions, with modifications. The modifications included; (i) the cell viability indicator (Green fluorescent dye; Ex. 483 nm and Em. 525 nm) was applied to separate wells from those receiving cell membrane dye (Red; Ex.562 nm and Em. 573 nm), both at a 1500 X dilution in PBS containing Ca, Mg (Sigma-Aldrich, St Louis, USA); and (ii) the cell membrane dye solution included a Hoechst 33,342 nuclear stain (1500 X dilution; Sigma-Aldrich) (Blue; Ex. 358 nm and Em. 463 nm). Medium was aspirated from the wells and 100 µL of each dye solution was added to each well and incubated at room temperature for 20 min. Thereafter, the dye was removed, and the background suppression dye added (at a 100X dilution in PBS with Ca, Mg; Sigma-Aldrich). PBS washes (3X) were included between each step to minimize substrate variability (Fig. 1 Step 2).

Quantification of relative fluorescence of the cell viability dye was carried out using the SynergyMx (Biotek) monochromator multi-fluorescence plate reader. Readings were captured using Gen5 software (v2.0). Cells were visualized using a Zeiss AxioVert.A1 FL microscope and images were captured at 200X magnification. Images were processed using Zen software 2011 (Blue ed.) and exported for the quantification of neurite outgrowth length using the default settings on the ImageJ plugin NeuriteTracer [23]. The NeuriteTracer analysis produced two values per image; the total neurite outgrowth and the nuclear count; which provided average neurite length per cell (mean ± SD).

### Cell Proliferation assay WST-1

To confirm changes in cellular proliferation following addition of RA, the WST-1 assay (Roche) was employed as per manufacturer’s instructions. Cells were seeded at 10 000 cells/well in a 96-well plate and allowed to settle for 24 h before being treated with RA at 10 µM. An untreated control and DMSO (0.01%) vehicle control wells were included for comparison. Following 96 h (Day 3) incubation, spent media was removed and replaced with 110 µL pre-warmed fresh basal media, containing 1:10 dilution (v/v) of WST-1 reagent and incubate for 2.5 h at 37 °C in a humidified CO_2_ incubator. Absorbance at 450 nm (reference wavelength of 650 nm) was determined using a SynergyMx multiplate reader (BioTek). The average of three consecutive readings were recorded. Wells containing media and WST-1 reagent only were used as a blank and subtracted from all data obtained. This was repeated for plates incubated to Day 6 and Day 9. Each experiment was carried out on three occasions, in duplicate (n = 6).

### Immunofluorescence staining

Cells were seeded on ethanol sterilized glass coverslips in 4-well culture plates (Nunclon^™^, Nunc, USA) and allowed to settle for 24 h prior to RA addition (Day 0). RA-containing medium was replenished on Day 3 and incubated until Day 6. Cells were fixed following aspiration of medium and washing with PBS (3 × 5 min); washed cells were incubated for 30 min in a 4% (w/v) formaldehyde solution at room temperature, followed by further washing in PBS (3 × 5 min) and permeabilization with 0.1% (v/v) Triton X. Prior to blocking, the coverslips were washed with PBS as before and incubated in 1% (w/v) bovine serum albumin (BSA)/PBS at room temperature for 1 h followed by an overnight incubation with relevant primary antibodies (in fresh 1% (w/v) BSA/PBS solution) at 4 ℃. Coverslips were washed (PBS; 3 × 10 min) before incubation with the relevant secondary antibody diluted in 1% (w/v) BSA/PBS in the dark at room temperature for an hour. Coverslips were washed (PBS, 3 × 10 min), before counterstaining with Hoechst 33,342 (1:1000 (v/v) diluted in ddH_2_0). A final wash step with PBS was performed prior to mounting on standard microscope slides using Dako fluorescence mounting medium (Dako, USA) prior to visualization using a Zeiss AxioVert.A1 fluorescence microscope and Zen Software 2011 (Blue Edition). Primary antibodies used included Nestin (10c2) mouse monoclonal IgG (1:600), Sox2 (H-65) rabbit polyclonal IgG (1:500), secondary labelled antibodies Alexa Fluor 488 Chicken anti-rabbit IgG (H + L) and Alexa Fluor 546 donkey anti-mouse IgG (both at dilution 1:500). Primary antibodies were purchased from Santa Cruz Biotechnology (Dallas, Texas, USA) and secondary antibodies were procured from Life Technologies (Carlsbad, California, USA).

### Statistical analysis

Statistical analysis was conducted using GraphPad Prism 4.0 (GraphPad software, Inc., California, USA). All experiments were conducted in technical and biological duplicates. Significance was considered as p < 0.05.

## Results and discussion

**Fig. 2:**
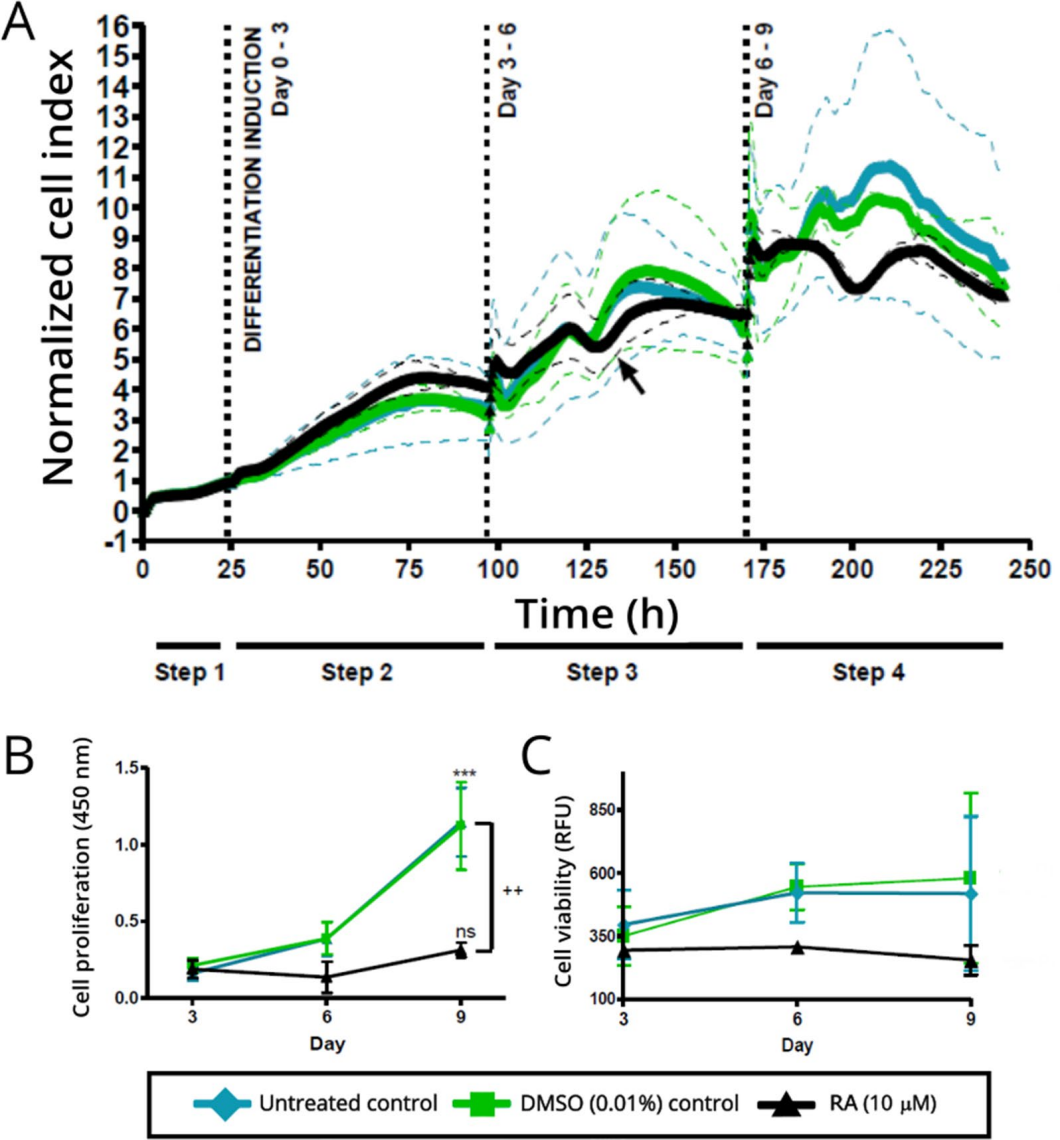
Characterization of SH-SY5Y retinoic acid-induced differentiation over a 9-day period. **A** First step xCELLigence analysis. Error bars are presented as dotted lines and are representative of n = 2 (each with quadruplicate replicates). All treatments were at 24 h post-seeding (Day 0; xCELLigence step 1), replenished on Days 3 and 6, and grown to Day 9 (xCELLigence step 2, 3 and 4 on xCELLigence software, respectively). Cell index curves were normalized to the first time point after compound addition. End-point analysis were conducted in replica 96-well plates **B** SH-SY5Y cells stop proliferating upon long-term RA treatment. WST-1 analysis depicting the change in cell proliferation/number over a period of 9 days of cell growth. The symbol *** denotes the intra-comparison between Days 3, 6 and Day 9 of both control groups (***p < 0.001) and ns (not significant; p > 0.05) of the RA-treated group. Inter-comparison between cell number at Day 9 between each treatment is denoted by the symbol + + where ( + + p < 0.01) **C** Cell viability was captured (Ex. 483 nm and Em. 525 nm) using the Synergy Mx multiplate reader. Error bars depict n = 3

Monitoring mammalian cellular differentiation using electrical cell substrate impedance sensing has been shown to be highly effective [12, 13, 17]. The workflow (Fig. 1) utilized the advantages of both non-invasive real-time analysis and classical end-point analysis to obtain data on cellular differentiation activity. A distinct pattern of a differentiating population, by observation of reduced cell index profile of the RA-treated cells (RA; 10 µM) relative to the untreated control (media only) and DMSO control (vehicle) is provided in Fig. 2A. The spikes and shifts (as shown by sudden shift in curve alignment) at the dotted lines at Day 3 and Day 6 are as a direct result of the full media volume replacement at these time points (Fig. 2A). A defined increase in cell index was observed until just before 150 h, with the Cell Index (CI) for the 10 µM RA treatment containing a definitive stationary phase, indicative of non-proliferative cells (Fig. 2A, arrow). This is echoed by the difference observed between proliferating cells in the proliferating controls (untreated and DMSO) and the terminally differentiated neurons observed following RA treatment (10 µM). This is further observed in the determination of the IC_50_ of RA (Additional file 1: Fig. S1). The distinction between proliferation, growth inhibition and proliferation are observable at 0–1 µM, 5–40 µM and 60–100 µM, respectively. At low concentrations, (0–1 µM) there is a clear increase indicative of continued proliferation; at 5–40 µM, we see a steady state reached indicative of a switch from proliferation to cessation of growth and finally at high concentrations (60–100 µM) we can observe characteristic cell death curves as shown by a rapid decrease in CI to baseline. In Fig. 2A, a CI increase is observed from initial induction of differentiation (marked as Differentiation Induction Day 0–3) which appears to indicate that cellular proliferation was still occurring during this time, as evidenced by the similar upward trend as observed between the RA treated cells and the untreated and DMSO controls. This is interesting as it does provide a view that during the time of differentiation induction, cellular populations may still be increasing at least in the initial phase (time point 25 to 50 h), this naturally is not observed in end point analysis. This may therefore be a combined effect of proliferation giving way to morphological changes associated with differentiation (50 to 100 h). Larger deviations between the proliferating controls (untreated and DMSO) are then observed from 100 h onwards. While patterns are similar during the Day 3–6 phase the largest deviations are observed following 150 h, i.e., between 175 and 225 h (Day 6–9 phase).

The xCELLigence provides a reproducible view of the distinct changes occurring in proliferating and differentiating cells over a prolonged 9-day period similar to those observed by Dwane et al. [16]. In the former study, differentiation was observed from the point of plating (t = 0) in the xCELLigence and differences in CI between differentiating and proliferating were immediately observed over a 24 h period. This may be ascribed to determining the measured difference between continued proliferation and migration and morphological changes, which are attributed to axonal guidance and neurite outgrowth during differentiation[16, 18]. Curiously, it has been reported that RA reduces the migratory and invasive ability of the parental SK-N-SK cell line which may further indicate that what was observed in the time period following initial addition of RA may indeed not entirely be proliferation events given the changes in CI observed fairly early in the programming by Dwane et al. [16, 24]. This is indeed confirmed by analysis of cell proliferation using the metabolic probe WST-1 shows that cell proliferation halts upon treatment of RA (Fig. 2B) relative to the controls. Further to this, no changes in cell viability were observed over the entire time course of RA treatment as measured by the Neurite Outgrowth Kit cellular viability fluorescence probe (Fig. 2C). It is therefore inferred that the differences observed between the controls (untreated and DMSO) and the RA treatments are largely due to the increased number of cells in the controls, relative to the changes in cell cycle that occur during differentiation i.e. halting of proliferation (Fig. 2B and C).

**Fig. 3:**
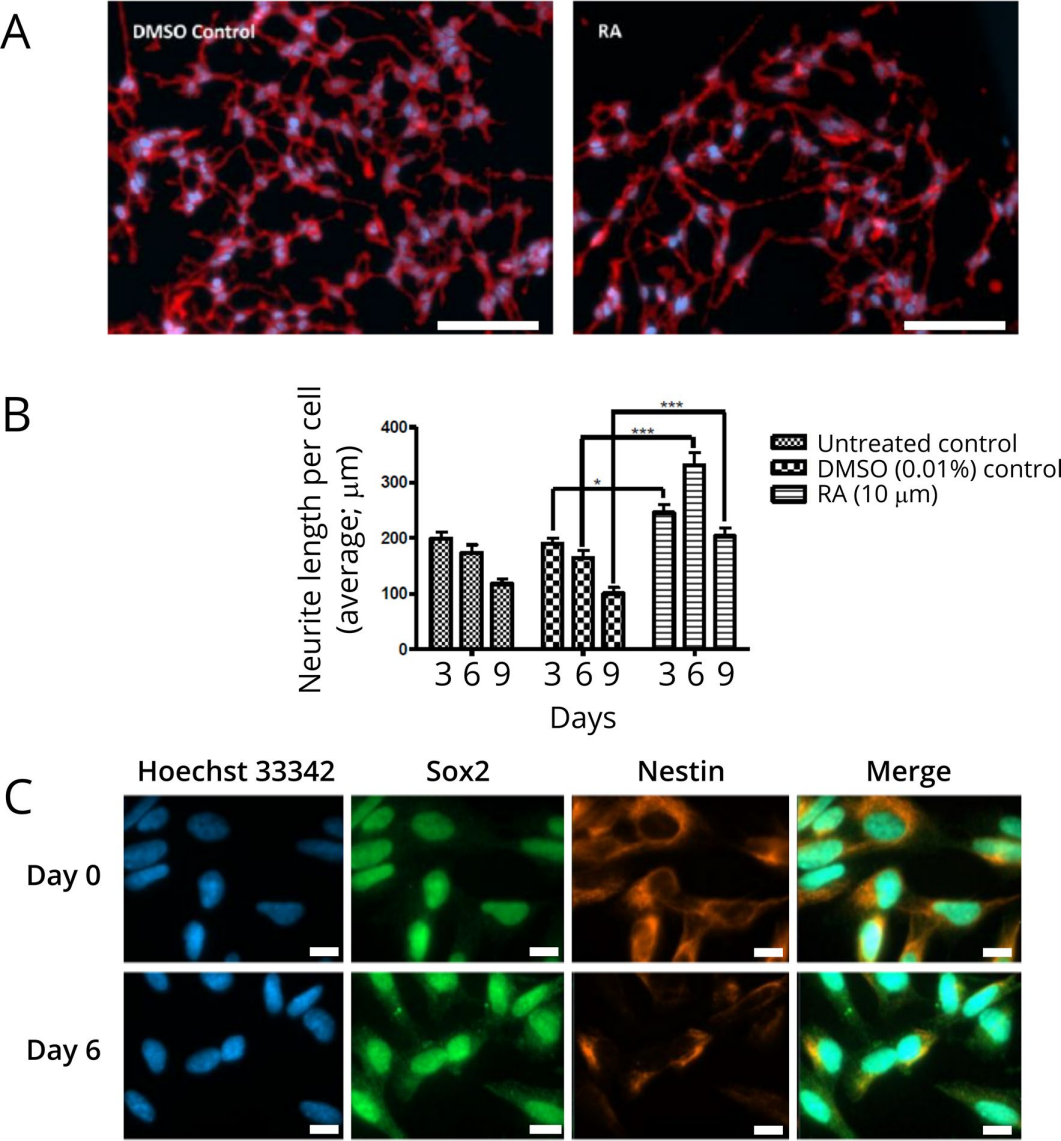
End-point quantitative and qualitative determination of differentiation **A** Representative fluorescence images captured for NeuriteTracer analysis. Images shown were captured on Day 3 post differentiation (shown, red = cell membrane dye in Neurite Outgrowth Staining kit and blue = Hoechst 33,342 nuclear counterstain). Scale bar = 100 μm. Experiments were carried out in duplicate **B** quantified using NeuriteTracer analysis (ImageJ). Statistical comparison of untreated and RA treated cells versus the DMSO-vehicle control on respective days (3, 6 and 9) was conducted using two-way ANOVA; *p < 0.05 and ***p < 0.001. RA-treated cells showed a significant increase in neurite length by day 9. **C** Immunofluorescence analysis of phenotype marker expression upon treatment with RA (10 µM) for 6 days. Images were captured using a Zeiss AxioVert.A1 FL-LED fluorescence microscope at 1000✕ magnification. Images are representative of multiple randomized fields of view images. Scale bar = 10 μm

This was visually confirmed by quantitative analysis of neurite outgrowth (Fig. 3A, B); where fewer cells were observed during differentiation (Fig. 3A, panel RA). Furthermore, the RA treatment (Days 3, 6 and 9 post treatment) resulted in an altered phenotype as evidenced by increased average neurite length per cell relative to the DMSO (0.01%) controls at Days 3 ,6 and 9 (Fig. 3B). To confirm changes to the molecular phenotype during prolonged treatment of RA, immunofluorescence staining for markers associated with neuronal differentiation, nestin and Sox2, were performed. Changes in the localization of the self-renewal marker Sox2, following 6 days post RA treatment (Fig. 3C, panel Sox2), and diffuse cytoplasmic staining (Day 6) relative to the nuclear localized Sox2 at Day 0 (Fig. 3C) was noted, indicating differentiation programming and lineage commitmen [25]. Fluorescence microscopy further revealed decreased expression of the biomarker nestin (Fig. 3C, panel nestin compare Day 0 to Day 6). A defined decrease in the cytoplasmic distribution of the intermediate filament protein nestin, which is associated with early development and is typically downregulated during SH-SY5Y neuronal differentiation, was noted [26].

In conclusion, RA-induced growth inhibition was identified by the xCELLigence analysis and supported by cell viability detection and observation of cellular proliferation. This is in accordance with observations by published literature where growth inhibition is a direct indication of onset of differentiation [18, 20]. Statistically significant changes in cellular morphology and neurite outgrowth length was identified by end-point fluorescence detection followed by computational analysis. Here we provide evidence that the ECIS platform can indeed monitor SH-SY5Y phenotypic differentiation (confirmed by end-point analysis) using the real-time monitoring technology employed paving the way for large scale application in screening of neurotrophic factor drug discovery. The distinctive ECIS CI patterns observed between the cellular phenotypes (i.e. undifferentiated and differentiated) acts a proof of concept that the system is sensitive enough to monitor neurite outgrowths over a prolonged time period within the constraints of traditional retinoic acid differentiation programming particularly in medium to high throughput and Hit-to-Lead screening.

## Limitations

The data presented here provides direct evidence of a link between differentiation and changes in impedance. This is however limited with respect to not providing a granular view of the relationship between impedimetric signal changes to specific morphological changes during neurogenesis.

## Supplementary information

**Additional file 1: Fig. S1.** Retinoic acid-induced cytotoxicity in the SH-SY5Y cell line is dose- and time-dependent.

## Data Availability

All the data supporting the study findings are within the manuscript. Additional detailed information and raw data are available from the corresponding author on reasonable request.

## Abbreviations

RA: Retinoic acid
ECIS: Electrical cell-substrate impedance sensing
RTCA SP: Real time cell analyser-single plate
DMSO: Dimethyl sulfoxide
CI: Cell index
PBS: Phosphate buffered saline
Ex: Excitation
Em: Emission
BSA: Bovine serum albumin
h: Hours
IgG: Immunoglobulin G
Sox2: SRY-box transcription factor 2
WST-1: water soluble tetrazolium salt
