# Supplementary material for: A simplified workflow with end-point validation of real-time electrical cell-substrate impedance sensing of retinoic acid stimulated neurogenesis in human SH-SY5Y cells in vitro
Source: BMC Res Notes. 2023 Jun 1;16:93. doi: 10.1186/s13104-023-06369-0 (PMC10233976; doi:10.1186/s13104-023-06369-0)
Supplement: Supplementary file 1 — Additional file 1: Fig. S1. Retinoic acid-induced cytotoxicity in the SH-SY5Y cell line is dose- and time-dependent. [file 13104_2023_6369_MOESM1_ESM.docx]

**A**


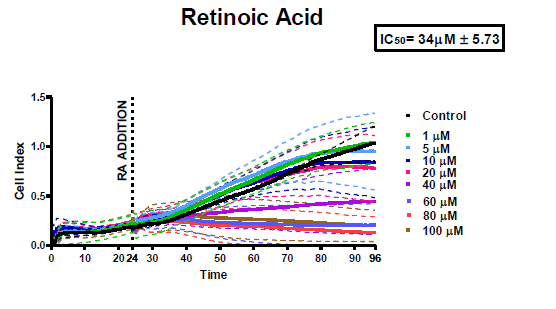


**B**

**Fig S1: Retinoic acid-induced cytotoxicity in the SH-SY5Y cell line is dose- and time-dependent.** The IC₅₀ (half-maximal inhibition) was calculated from (A) real-time data collected from the xCELLigence RTCA SP. (B)The IC₅₀ value was determined using the xCelligence developed graph of Cell Index (CI) vs. log of concentration (M), where a sigmoidal dose-response curve (variable slope) was generated. The time period consisted of 72 hours, from the point of compound addition (depicted by dotted line shown in A); this excluded the initial 24-hour time period allowed for cell settling. The IC₅₀ (µM) was calculated and is shown above, with the average ± SD (n =2) and average R² value of 0.98.
